# Machine learning-aided analysis for complex local structure of liquid crystal polymers

**DOI:** 10.1038/s41598-019-51238-1

**Published:** 2019-11-08

**Authors:** Hideo Doi, Kazuaki Z. Takahashi, Kenji Tagashira, Jun-ichi Fukuda, Takeshi Aoyagi

**Affiliations:** 1 Research Center for Computational Design of Advanced Functional Materials, National Institute of Advanced Industrial Science and Technology (AIST), Central 2, 1-1-1 Umezono, Tsukuba, Ibaraki 305-8568 Japan; 2 Research Association of High-Throughput Design and Development for Advanced Functional Materials, Central 2, 1-1-1 Umezono, Tsukuba, Ibaraki 305-8568 Japan; 3 Department of Physics, Faculty of Science, Kyushu University, 744 Motooka, Nishi-ku, Fukuoka, Fukuoka, 819-0395 Japan

**Keywords:** Condensed-matter physics, Nanoscale materials, Soft materials, Techniques and instrumentation, Theory and computation

## Abstract

Elucidation of mesoscopic structures of molecular systems is of considerable scientific and technological interest for the development and optimization of advanced materials. Molecular dynamics simulations are a promising means of revealing macroscopic physical properties of materials from a microscopic viewpoint, but analysis of the resulting complex mesoscopic structures from microscopic information is a non-trivial and challenging task. In this study, a Machine Learning-aided Local Structure Analyzer (ML-LSA) is developed to classify the complex local mesoscopic structures of molecules that have not only simple atomistic group units but also rigid anisotropic functional groups such as mesogens. The proposed ML-LSA is applied to classifying the local structures of liquid crystal polymer (LCP) systems, which are of considerable scientific and technological interest because of their potential for sensors and soft actuators. A machine learning (ML) model is constructed from small, and thus computationally less costly, monodomain LCP trajectories. The ML model can distinguish nematic- and smectic-like monodomain structures with high accuracy. The ML-LSA is applied to large, complex quenched LCP structures, and the complex local structures are successfully classified as either nematic- or smectic-like. Furthermore, the results of the ML-LSA suggest the best order parameter for distinguishing the two mesogenic structures. Our ML model enables automatic and systematic analysis of the mesogenic structures without prior knowledge, and thus can overcome the difficulty of manually determining the specific order parameter required for the classification of complex structures.

## Introduction

Recent advances in computer power and simulation techniques make it possible to perform large-scale molecular simulations of macromolecules. However, analysis of the resulting complex mesoscopic structures from microscopic information is a non-trivial and challenging task, especially for amorphous, glassy, liquid-crystalline, and quenched materials. For mass point or spherical particle systems, several analysis methods that focus on the arrangement of particles have been widely accepted^1^. These methods are based on particle coordinates and can classify many types of molecular forms, such as liquid, solid, body-centered cubic, face-centered cubic, hexagonal close-packed, and vacancy structures. These have been applied to many different problems: classifying the phases of Lennard–Jones (LJ) systems^2^ or water systems^3^, drawing the phase diagram of confined LJ systems^4^, observing the crystal nucleation of molten Cu^5^, observing the spontaneous nucleation and growth of methane hydrate^6^, and detecting the vacancy and interstice of many types of molecular structures^7–9^. For anisotropic rigid particle systems such as liquid crystals, however, the above analyses are not easy because of the additional orientational degrees of freedom. For instance, the aspect ratio of the mesogenic ellipsoids, which is determined for specific problems and therefore user-dependent (i.e., by changing the chemical architecture of mesogens in experiments), significantly affects the structures and phases of the system. Difficulties in predicting the structures and phases prior to the simulations preclude preliminary development of specific order parameters that classify the system structure. For bulk liquid crystalline systems, classification of phases is not difficult because of a good performance of well-established order parameters such as Onsager order parameter^10^ and McMillan order parameter^11^. For complex systems with nonuniform local structures of liquid crystals, however, it is non-trivial whether the above order parameters maintain their performance or not. Therefore, it is desirable to develop a systematic and automatic classification method that does not require prior knowledge of the structures. Recently, Spelling and co-workers demonstrated how machine learning (ML) can be applied to discovering interesting areas of parameter space which are closely related to characteristic colloidal structures^12^. Carrasquilla and co-workers demonstrated that ML using the convolutional neural network (NN) can detect topological phases that cannot be classified by conventional order parameters^13^. VanNieuwenburg and co-workers proposed a ML approach to find phase transitions from the performance of the NN after training it with deliberately incorrectly labeled data^14^. Rodriguez and co-workers propose an unsupervised approach based on diffusion maps that learns topological phase transitions from raw data without the need of manual feature engineering^15^. Walters and co-workers demonstrated supervised machine learning can classify topological defects in a two dimensional confined liquid crystal system^16^.

Effective descriptors were designed from numerical fingerprints of structures found in colloidal self-assembly, and were applied to both automatically finding interesting areas on a phase diagram and automatically identifying important parameter groups related to simple/complex local crystal structures. Their study demonstrates the significant potential of ML for automatic classification and discovery of molecular structures.

Here we developed a ML-aided Local Structure Analyzer (ML-LSA) for classifying complex local mesoscopic structures of molecules that are composed of not only isotropic particles but also anisotropic rigid functional groups such as mesogens. The proposed ML-LSA was applied to classifying the local structures of liquid crystal polymer (LCP) systems.

There are two major reasons why we chose LCPs as the target of our ML-LSA. One is that liquid crystal networks (LCNs) that consists of LCPs have been attracting growing interest as a promising material for practical applications such as sensing and actuator devices; LCNs exhibit macroscopic deformations to modest external stimuli such as electrical and magnetic fields and irradiated light^17–26^, which is attributable to the combination of the orientational degrees of freedom and the entropic elasticity of the polymer network and the resulting soft elasticity. Indeed this unique responsiveness motivated the development of several types of LCN actuators^27–29^.

The other reason is the difficulty in the characterization of local structures of LCPs obtained by particle-based simulations. Modeling liquid crystal molecules as coarse-grained anisotropic particles has been regarded as a promising approach for predicting micro- and mesoscopic properties of LCPs^30–34^. In previous studies, “voxel” analysis has conventionally been employed for structure analysis (e.g., evaluation of the orientational tensor order parameter^35,36^ and visualization of topological defects^37–41^), in which the simulation box is split cuboidal voxels each containing a single mesogenic particle on average. Each local structure is defined by grouping neighboring voxels that contain mesogenic particles oriented in the same direction. However, the local structure determined by voxel analysis could depend on how the simulation box is split into voxels, although voxels are introduced just for analysis purposes and do not have any physical meaning on their own. Note also the difficulty of voxel analyses for a deformable system (indeed the deformation of LCNs in response to external stimuli is an important research subject as already mentioned). Voxels of fixed position and shape cannot trace the deformation of the system. On the other hand, when the voxels are deformed so that they conform to the deformation of the whole system, deformed voxels may not be able to capture the local properties of the systems when their aspect ratio deviates significantly from 1. Therefore, it is highly desirable to develop analysis methods without relying on artificial voxels.

The ML-LSA proposed in this work was applied to our own MD simulations of LCPs using the SCGB (soft-core Gay-Berne) model similar to what Skačej and Zannoni developed for their Monte-Carlo simulations^32^. To construct a ML model which classifies “smectic-like” and “nematic-like” structures of LCPs, we first obtained small and thus computationally less demanding monodomain LCP trajectories. Next, to simulate the microscopic structure change induced by temperature variations, LCP trajectories for a larger system were quenched from the temperature condition for the isotropic structure to that for the smectic-like structure. The quenched trajectories generally have complex local structures that contain nematic- and smectic-like mesogenic structures; however, conventional particle-based analysis cannot distinguish between the two. Using ML-LSA, we successfully classified these complex local structures, regardless of the choice of user-dependent parameters, i.e., the aspect ratio of mesogenic rigid ellipsoids. Furthermore, the results provided by ML-LSA suggest the best order parameter for distinguishing between nematic- and smectic-like mesogenic structures. Our ML model enables automatic and systematic analysis of the mesogenic structures without prior knowledge, and thus can overcome the difficulty of manually determining the specific order parameter required for the classification of complex structures.

## Methodology

### Machine learning-aided local structure analyzer

**Figure 1:**
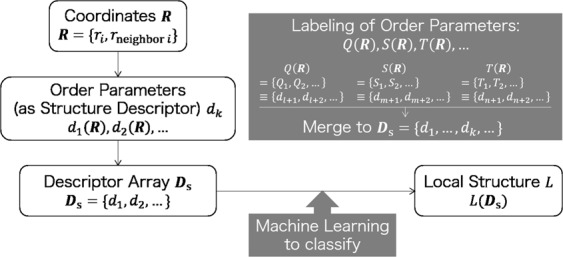
Conceptual image of ML-LSA, where *r*_*i*_ is the coordinate of particle *i*, *r*_neighbor*i*_ is the coordinates of particles in the neighborhood of *i*, and the terms *Q*, *S*, and *T* are examples of equations that give the order parameters (see Supporting Information).

ML is a powerful technique for analyses such as classification, interpolation, and regression. To find out unknown information, ML is generally applied without prior knowledge. To describe some potential characteristics of the data, a set of numerical values called a “descriptor” is defined and used. The descriptor should capture the characteristics of the data for the specific problem. Figure 1 shows a conceptual image of ML-LSA. In this study, we defined order parameters as structure descriptors, and defined an array *D*_s_ as the set of order parameters. To define this structure descriptor, eleven equations related to modified or developed order parameters were employed: the local Onsager order parameter^10^ *S*; local McMillan order parameter^11^ *T*; bond order parameter^42^ *Q*; common neighborhood parameters^43^ *A*, *P*, and *M*; bond angle analysis^44^ *B*; centrosymmetry parameter analysis^45^ *C*; neighbor distance analysis^46^ *D*; angular Fourier-series-like parameter^47,48^ *F*; and the angle histogram analysis parameter *H* (details of each order parameter are given in the Supporting Information). Importantly, the exact form of each order parameter changes with the setting of variables such as the number of neighboring particles (see Table S1 in Supporting Information). These variables ensure that the order parameters have different sensitivities for describing the characteristics of the data. Therefore, we individually counted and labeled the order parameters given by the same equation but different variables. Using this labeling rule, the number of order parameters increased to almost 1,600,000 (see Table S1 in Supporting Information). The set of order parameters was merged to form the array of structure descriptors *D*_s_. To classify the local structure of quenched LCP trajectories, we performed three ML steps. Firstly, each component of the structure descriptor (i.e., each order parameter) was calculated for well-established single-domain nematic- and smectic-like trajectories of LCP systems. Note that the order parameters were calculated for each mesogen particle *i* to show the local structure around *i*. The structure descriptor for each particle was labeled as either “nematic-like” or “smectic-like” for a supervised classification done at the next step. Next, to obtain the ML model for each order parameter, a supervised classification was performed using the random forest technique^49^. Note that the ML model (or the hypothesis) is a certain function that strongly resembles the target function, which is expected to clearly express a certain proposition. More specifically, each obtained ML model can be expected to describe well the characteristics of each order parameter for detecting nematic- or smectic-like structures. Thus, when the ML model performs well, the classification accuracy for distinguishing between single-domain nematic- and smectic-like structures becomes high. Finally, we applied the above ML model to classify the local structures of quenched LCP trajectories. Quenched trajectories themselves are extremely hard to use to obtain ML model because of the complex mixture of nematic- and smectic-like local structures. Thus the ML model determined from well-established single-domain trajectories were used, and mainly evaluated from comparison with several conventional classification methods.

Note that we do not use NN, just use random forest. For two-dimensional problems, the NN has proven to be a powerful method for machine learning: it does not need order parameters for classifying some kinds of phases/structures. However, from the “curse of dimensionality”, there is a high possibility that the NN becomes computationally expensive for three dimensional problems. Therefore, we developed the combination of supervised ML and order parameters rather than using NN.

### Molecular dynamics simulations

**Figure 2:**
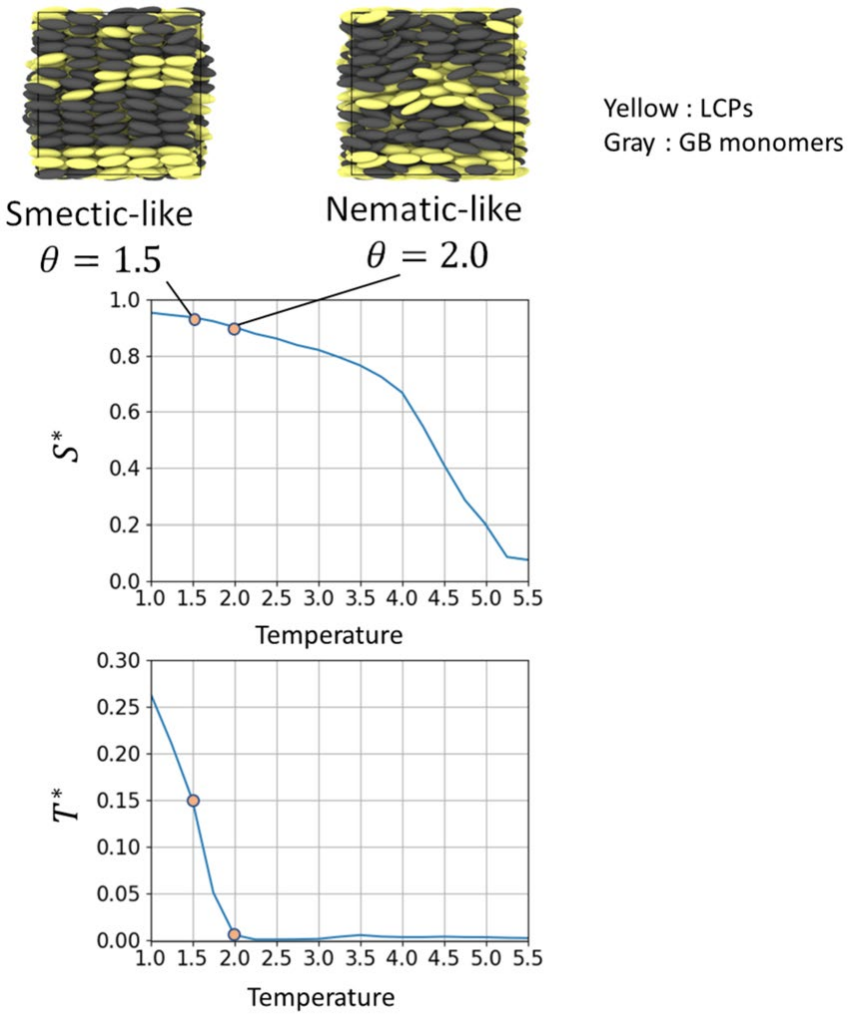
Temperature dependence of Onsager order parameter S^*^ and McMillan order parameter T^*^ for small LCP systems.

We performed coarse-grained MD simulations for LCPs using the SCGB model^32^. The units and dimensions of the values introduced below are exactly the same as in ref.^32^. The simulations were performed using constant particle-number, volume, and temperature conditions. The monomer density of the GB particle *ρ* was set to 0.3. The velocity Verlet integrator^50^ was used with three-dimensional periodic boundary conditions and a time step of 0.01. The LCPs were expressed by the combination of 5-mer GB main chains, and the monomer density of LCPs was fixed to 0.15. The LCP systems were swelled by monomeric GB particles, and the density of swelling GB particles was fixed to 0.15 (*i.e*., a degree of swelling was kept to 50%). In this study, two types of LCP systems were considered: (i) small systems for obtaining the ML model, (ii) large systems for simulating the microscopic structure changes induced by temperature changes. For small systems, the equilibrated trajectories of 972 GB particles were computed under temperature conditions of *θ* = 1.5 and 2.0 to form smectic- and nematic-like structures, respectively (see Fig. 2 in next section). The ML model was constructed from the trajectory given by a system of GB particles with aspect ratios of 3.0. The accuracy of the model was then examined through the classification of structures for systems of GB particles with aspect ratios of 2.8, 3.0, and 3.2. For large systems, GB particles with aspect ratios of 3.0 were used. The equilibrated isotropic structures of 62,204 GB particles at *θ* = 5.5 were gradually quenched to the condition *θ* = 1.5 over 100,000 simulation steps.

## Results and Discussion

### Machine learning using monodomain lcp systems

Figure 2 shows the temperature dependence of the original Onsager order parameter S^*^ and the original McMillan order parameter T^*^ for small LCP systems. The former is defined by the following equation,

1 $${S}^{\ast }=\frac{1}{{N}_{{\rm{s}}ys}}\mathop{\sum }\limits_{i}^{{N}_{{\rm{s}}ys}}\,\{3/2{(n\cdot {u}_{i})}^{2}-1/2\},$$

where the *N*_sys_ is the number of GB particles in the system, the *n* is the director which denotes the average of orientation direction of GB particles, and the *u*_*i*_ is the unit vector along the long axis of the GB particle *i*. The definition of T^*^ is

2 $${T}^{\ast }=\frac{{\sum }_{i}^{{N}_{{\rm{sys}}}}\,\cos (2\pi z/d)\{3/2{(n\cdot {u}_{i})}^{2}-1/2\}}{{N}_{{\rm{sys}}}},$$

where the *z* is the distance between GB particle *i* and the closest smectic-A layer, and the *d* is the distance between two smectic-A layers. Snapshots of trajectories were also shown for temperature conditions of *θ* = 1.5 and 2.0. For *θ* = 1.5, the monodomain smectic-like structure was observed. For *θ* = 2.0, the monodomain nematic-like structure was observed. The difference of two structures was visually clear, but there was almost no difference of Onsager order parameters S^*^ for the two. In contrast, McMillan order parameter T^*^ can distinguish the two. However, it is non-trivial whether this order parameter maintain its performance for complex systems that consist of local structures of LCPs. In particular, polymeric networks consisting of LCP chains may let local structures of mesogens freeze at untypical pattern. Therefore, we developed the ML-LSA for systematic and automatic classification that is independent of knowledge of the structures, and applied it for LCP systems.

To obtain the ML model for each order parameter, ML was performed for the small monodomain nematic- and smectic-like trajectories. The “accuracy” of each order parameter was defined as the correct answer rate to the following question: To which structure does the given trajectory belong? The correct answer rate *X* was defined as follows,

3 $$X=\frac{{Z}_{{\rm{correct}}}}{{Z}_{{\rm{total}}}},$$

where the *Z*_correct_ is the number of correct answers to the above question and the *Z*_total_ is the total number of the answers. Finding single order parameter with high *X* is one of our purposes in this work, because the high *X* means that the histogram of value of the order parameter can be clearly divided into two areas respectively showing nematic- and smectic-like structures. Note that the large amount of answers should be required to ensure the accuracy of the histogram itself. Otherwise, the ML results may suggest the order parameter with high *X* only at a specific situation.

**Figure 3:**
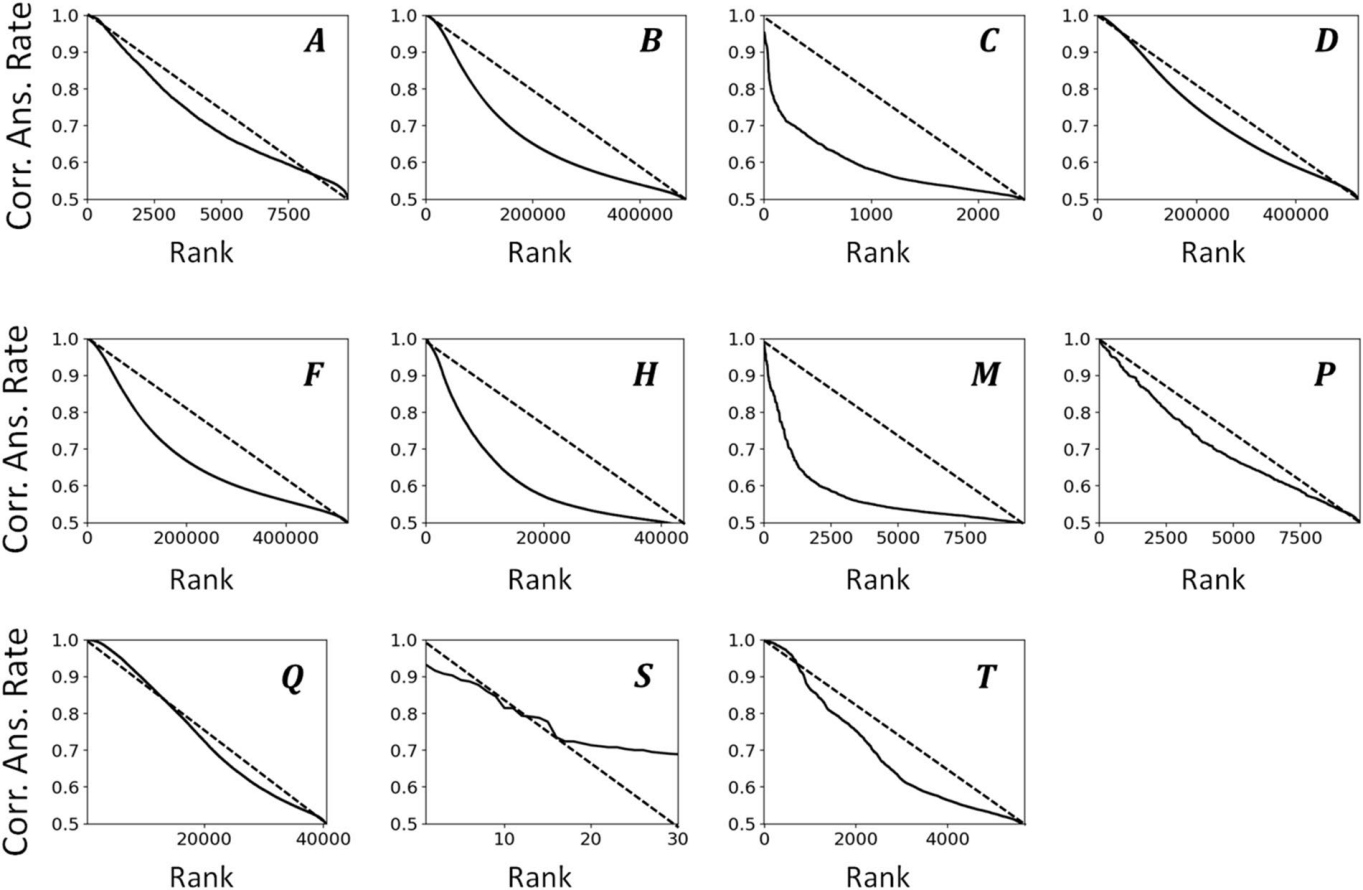
Correct answer rate of order parameters. The results are plotted with respect to each equation that gave the same type of order parameter. The *x*-axis of each graph represents an identification number of each order parameter sorted with respect to the correct answer rate. The results are shown in three different graphs because they can be separated into three tendencies.

Figure 3 shows the correct answer rate of each order parameter. Note that the results were plotted with respect to each equation giving the same type of order parameter. The *x*-axis of each graph represents a ranking of each order parameter, which is sorted with respect to the correct answer rate. The dashed line connects the ideal highest accuracy point (rank = 1, *X* = 1) with the lowest accuracy point (bottom rank, *X* = 0.5). As mentioned in Section 2.1, the exact form of each order parameter changes with the setting of variables. A simple example is the local McMillan order parameter *T* that depends on the variable *d* representing the distance between two smectic-like layers. Changing *d* obviously affects the performance of *T*. The description of other variables for order parameters are shown in Table S1 in Supporting Information. These variables ensure that the order parameters have different sensitivities for describing the characteristics of the data. Figure 3 draws the performance of each order parameter. The high correct answer rate indicates few overlapping of the histogram of order parameter values that reflect smectic-like or nematic-like local structures. To help the understanding, the data of order parameter *S* plotted on the Fig. 3 are displayed in Table 1 as the example. The results sorted by the rank indicate the tendency of the accuracy for each type of order parameter. Focusing on the peak performance of each type of order parameter (i.e., the results at the left side of each graph), all parameters except *S* can accurately distinguish between the two structures. However, the distribution of the correct answer rate varies among the order parameters. For order parameters *B*, *C*, *F*, *H*, and *M*, the accuracy decreases exponentially with the increase of rank, indicating that it is relatively difficult to stably produce good order parameters for various conditions of input data such as coordinates of particles. For order parameter *S*, the accuracy decreases slowly with the increase of rank, however, the highest performance of *S* is quite low of 0.93. Order parameter *Q* displayed the best performance, attaining both high peak performance and a slow decay of accuracy with respect to the *x*-axis. This slow decay indicates the robustness of this order parameter for local structure analyses. Namely, *Q* attained to stably produce good order parameters for various conditions of input data.

**Table 1:** Correct answer rate of the local Onsager order parameter *S*, where *N* is the number of neighborhood particles, *R* is the radius of the neighborhood, and *a* is the number of times of averaging the values of order parameter that particles neighboring the *i*-th particle have. Note that *N* is equal to the size of an array storing the identification numbers of particles neighboring the *i*-th particle, *a*.

Rank	Neighborhood condition	Condition *a*	Correct answer rate
1	*R* = 3.00	2	0.931
2	*N* = 27	2	0.916
3	*R* = 2.75	2	0.908
4	*N* = 24	2	0.903
5	*N* = 20	2	0.890
6	*R* = 2.50	2	0.886
7	*N* = 16	2	0.877
8	*R* = 2.25	2	0.860
9	*N* = 12	2	0.846
10	*R* = 2.00	2	0.814
11	*N* = 8	2	0.814
12	*N* = 5	2	0.793
13	*N* = 6	2	0.791
14	*R* = 1.75	2	0.788
15	*N* = 4	2	0.777
16	*R* = 3.00	1	0.735
17	*R* = 2.75	1	0.724
18	*N* = 27	1	0.723
19	*N* = 24	1	0.718
20	*N* = 20	1	0.713
21	*R* = 2.50	1	0.711
22	*R* = 2.25	1	0.708
23	*N* = 12	1	0.708
24	*N* = 16	1	0.703
25	*R* = 2.00	1	0.700
26	*N* = 6	1	0.700
27	*N* = 5	1	0.695
28	*N* = 8	1	0.693
29	*R* = 1.75	1	0.690
30	*N* = 4	1	0.689

The *R* is an alternative expression of neighborhood using distance from *i*, but generally corresponds to *N*. The description for the *a* and equation of *S* are shown in Supporting Information.

**Figure 4:**
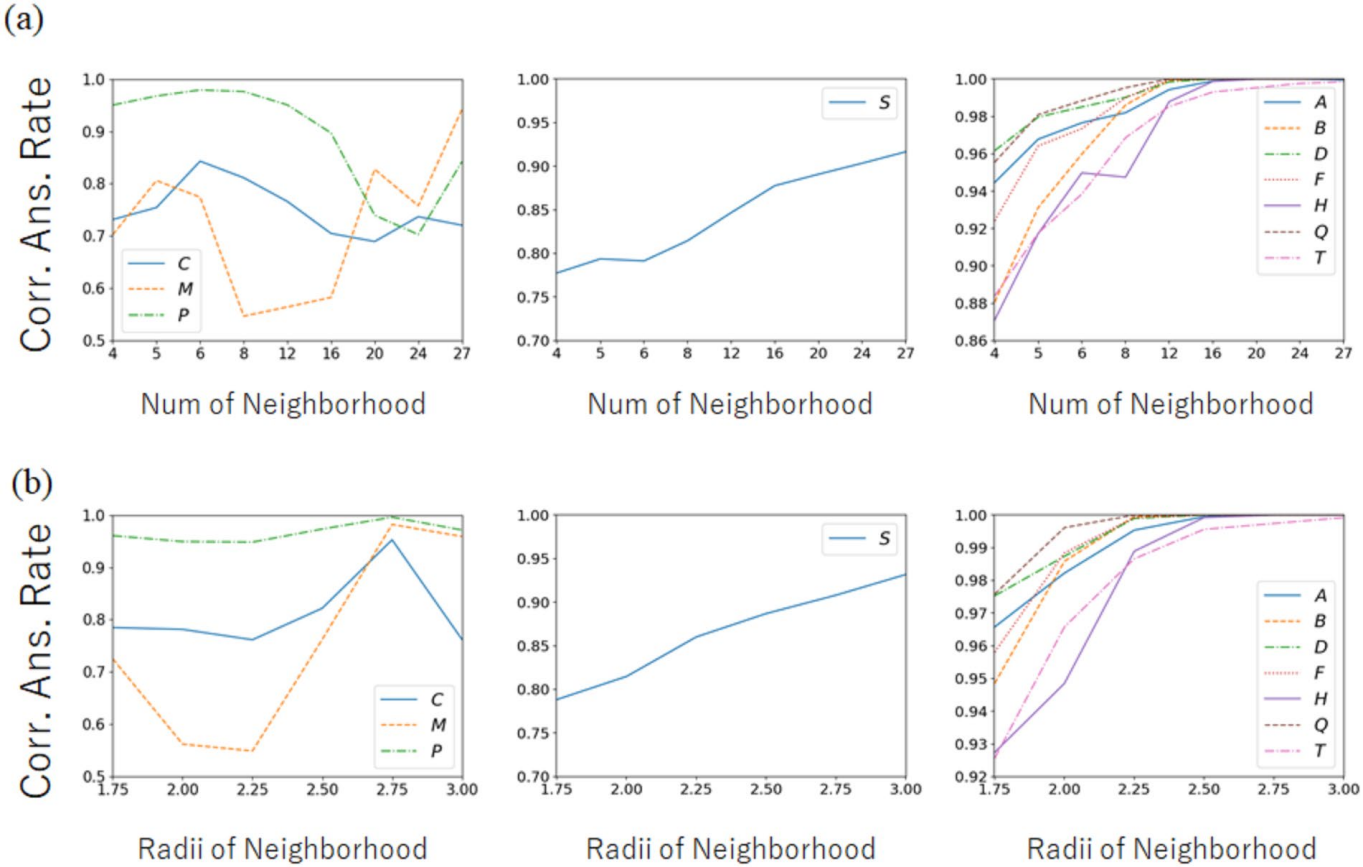
Highest accuracy of each type of order parameter with respect to (**a**) the number of neighborhood particles, and (**b**) the radius determining the neighborhood. The results are shown in three different graphs because they can be separated into three tendencies.

The accuracy of each order parameter can be affected by the definition of the neighborhood of particle *i*. For good order parameters, their accuracy is expected to be stable and high with respect to variations of neighborhood information. Figure 4 shows the highest accuracy of each type of order parameter with respect to the number *N* of neighborhood particles and the radius *R* of the neighborhood. The results can again be separated into three tendencies. For *C*, *H*, and *P*, the accuracy is unstable. Each parameter attained its peak performance under a certain condition, but the accuracy did not consistently increase along with the increment of information in the neighborhood. This instability becomes a bottleneck for accurate analyses. For *S*, a consistent increment in accuracy corresponding to the neighborhood information can be observed, but the accuracy itself is generally low. For the others, the accuracy is stable and increases monotonically with respect to the neighborhood information. The origin of the instability of *C*, *H*, and *P* can be thought of as the similarity of the equations; however, *A* did not exhibit such instability despite having a similar equation to the above three parameters. *Q* attained the ideal correct answer rate (=1) under the condition *N* ≥ 12 or *R* ≥ 2.25. Note that the above tendencies of the order parameters only apply to LCP systems. It is possible that completely different tendencies would be observed for analyses of other materials. For ML, any prediction of the capability of order parameters using previous knowledge should be avoided.

**Figure 5:**
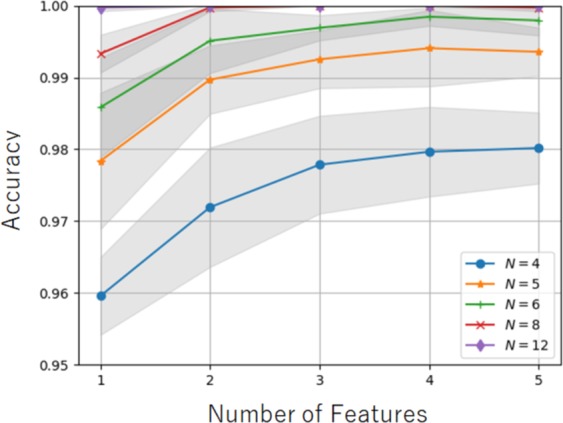
Correct answer rate (i.e., accuracy) for combination of features selected by SFS. The accuracy increases with respect to *N* and the number of features. For *N* = 4, 5, and 6, an increase in the number of features up to four effectively improves the accuracy. The addition of the fifth feature does not improve the results. For *N* = 8, the accuracy is saturated with two or more features. For *N* = 12, the accuracy is almost ideal regardless of the number of features. The condition *N* = 8 with two features and the condition *N* = 6 with four features is close to the accuracy of the condition *N* = 12.

**Figure 6:**
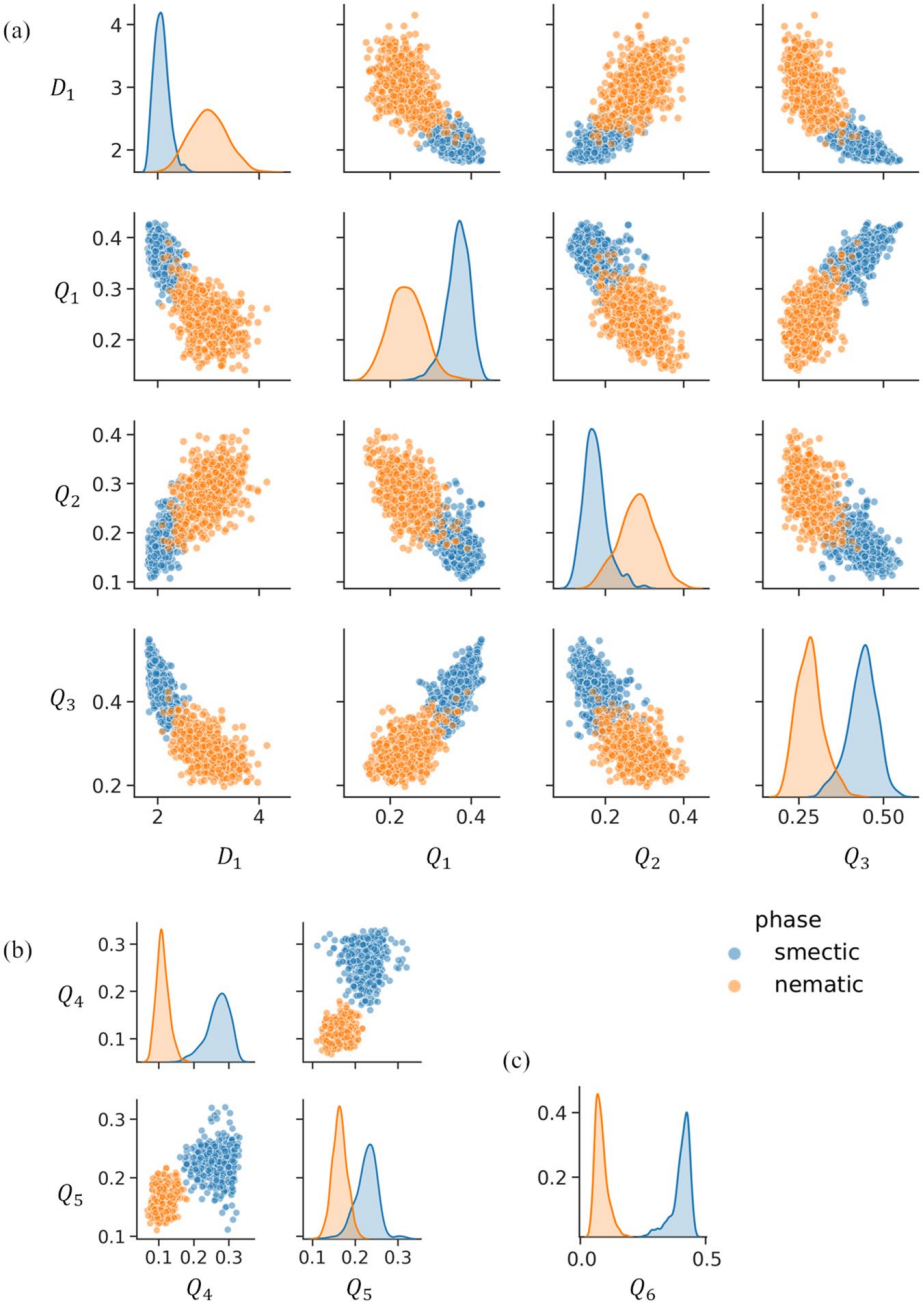
Results of SFS illustrated by pair-plots of features under conditions (**a**) *N* = 4 with four features, (**b**) *N* = 8 with two features, and (**c**) *N* = 12 with one feature. The blue and yellow dots represent particles in smectic- and nematic-like trajectories, respectively. For *N* = 4, the data for all pair-plots slightly overlap, indicating that one or two features cannot completely distinguish between the nematic- and smectic-like structures. For *N* = 8, the data for the pair-plots almost separate into two regions, suggesting good performance by the combination of two features. In contrast, the data for one feature are slightly overlapped. For *N* = 12, the data for the one feature are completely separated into two fields. The definitions of the order parameters *D*_1_, *Q*_1_, *Q*_2_, *Q*_3_, *Q*_4_, *Q*_5_, and *Q*_6_ are shown in the Supporting Information.

By using a certain amount of information for the monodomain local structures, we have shown that a single feature (i.e., a single order parameter) can attain a high correct answer rate for distinguishing between the two structures. For higher computational efficiency, however, the reduction of local information, such as the value of *N*, should be examined. To attain a suitable level of accuracy while reducing *N*, the selection of an efficient combination of features is a promising approach. However, choosing components of the efficient combination is non-trivial task because the results of random forest itself do not suggest any information for relationships among features. A sequential forward selection (SFS) algorithm^51^ is one possible way to identify high-performance feature sets for classification using random forest classifier. In SFS, one feature is firstly selected, which displays the best performance on the single feature ML model. Then the second feature is selected, which displays the best performance on the two feature ML model by combining with the first feature. The third and subsequent features can be sequentially selected the same manner, until the performance is saturated. From this sequential selection, the small set of effective features can be determined. The SFS was performed for the results of random forest. All of the order parameters were prepared for the nematic- and smectic-like trajectories under the neighborhood conditions *N* = 4, 5, 6, 8, 12. SFS was then performed to decrease the large number of order parameters to five features. Figure 5 shows the correct answer rate for the combination of features selected by SFS. Overall, the accuracy increased with respect to *N* and the number of features. For *N* = 4, 5, and 6, an increase in the number of features up to four was effective in improving the accuracy. The addition of the fifth feature did not improve the results. For *N* = 8, the accuracy was saturated with two or more features. For *N* = 12, the accuracy was almost ideal regardless of the number of features. The condition *N* = 8 with two features and the condition *N* = 6 with four features came close to the accuracy of the condition *N* = 12. Figure 6 visualizes the results of SFS through pair-plots of features under the conditions *N* = 4 with four features, *N* = 8 with two features, and *N* = 12 with one feature. For *N* = 4, the data for all pair-plots slightly overlap, indicating that one or two features could not completely distinguish between the nematic- and smectic-like structures. Note that the accuracy of this condition reached 0.98 using all four features. Importantly, *D*_1_ was selected as an efficient feature together with *Q*_1_, *Q*_2_, and *Q*_3_. This shows that the combination of different types of order parameters can improve the accuracy of classification. For *N* = 8, the data for the pair-plots almost separate into two regions, indicating good performance by this combination of features. In contrast, the data for a single feature only slightly overlap. This means that the combination of two features, *Q*_4_ and *Q*_5_, improved the accuracy. For *N* = 12, the data for the single feature completely separate into two fields. This means that *Q*_6_ is an excellent feature for classification. Overall, *Q* produced excellent order parameters when selected as features.

Note that the above ML models and analysis results are for systems of GB particles with aspect ratios of 3.0. For GB particles with aspect ratios of 2.8 or 3.2, however, the analysis results using the same ML models are almost the same as those for GB particle with aspect ratios of 3.0 (data not shown). This indicates that the ML models are not significantly influenced by the aspect ratio over the range 2.8–3.2.

### Local structure analysis for complex lcp systems

**Figure 7:**
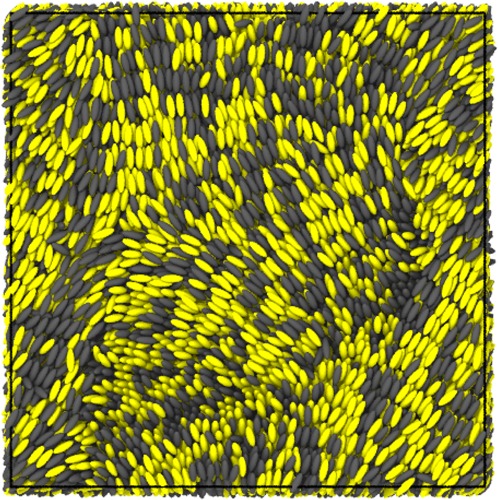
Snapshot of complex trajectories made by quenching from the temperature condition of the isotropic structure (*θ* = 5.5) to that of the smectic-like structure (*θ* = 1.5). The yellow particles belong to LCPs, and the gray particles are LC molecules for swelling. The trajectories exhibit complex local structures, but it is difficult to visually classify each local structure.

**Figure 8:**
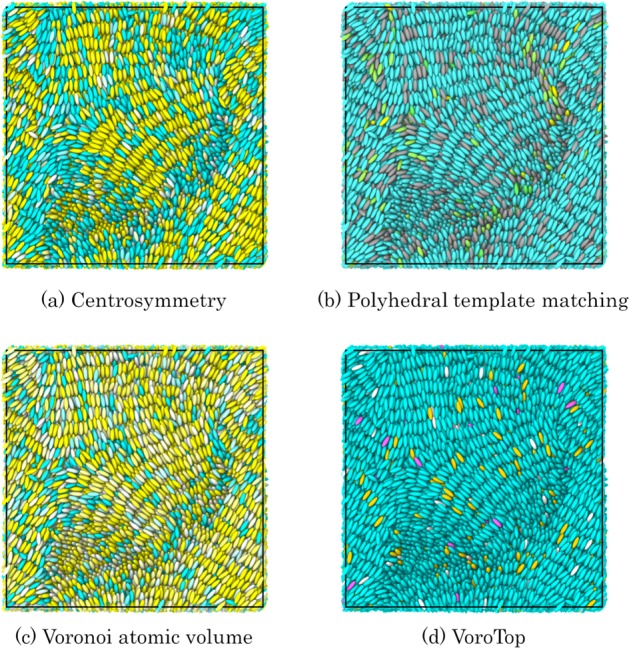
Results of conventional particle-based local structure analyses. (**a**) The centrosymmetry method with 12 neighboring particles. The particle colors gradually change from yellow to light blue with respect to the change in the centrosymmetry parameter from 3 to 4. (**b**) The polyhedral template matching method with a 0.6 root-mean squared cutoff. The yellow color denotes a simple cubic structure, and the blue color represents other structures. (**c**) The Voronoi atomic volume method implemented on Ovito. The particle colors gradually change from yellow to light blue as the volume of the Voronoi cells increases from 3 to 4. (**d**) The Voronoi cell topology visualization and analysis toolkit (VoroTop). The pink color denotes a hexagonal close-packed structure, and the blue color represents other structures. None of the methods could distinguish any local structures of ellipsoidal rigid mesogenic systems.

To obtain complex LCP trajectories, systems were quenched from the temperature condition of the isotropic structure (*θ* = 5.5) to that of the smectic-like structure (*θ* = 1.5). Figure 7 shows a snapshot of some complex trajectories. The trajectories have complex local structures, but it is difficult to visually classify each local structure. First, we examined four conventional particle-based analysis methods: centrosymmetry^45^, polyhedral template matching^52^, Voronoi atomic volume, and the Voronoi cell topology visualization and analysis toolkit (VoroTop)^7^. The results are shown in Fig. 8. It is clear that the four conventional methods cannot distinguish any local structures of ellipsoidal rigid mesogenic systems.

**Figure 9:**
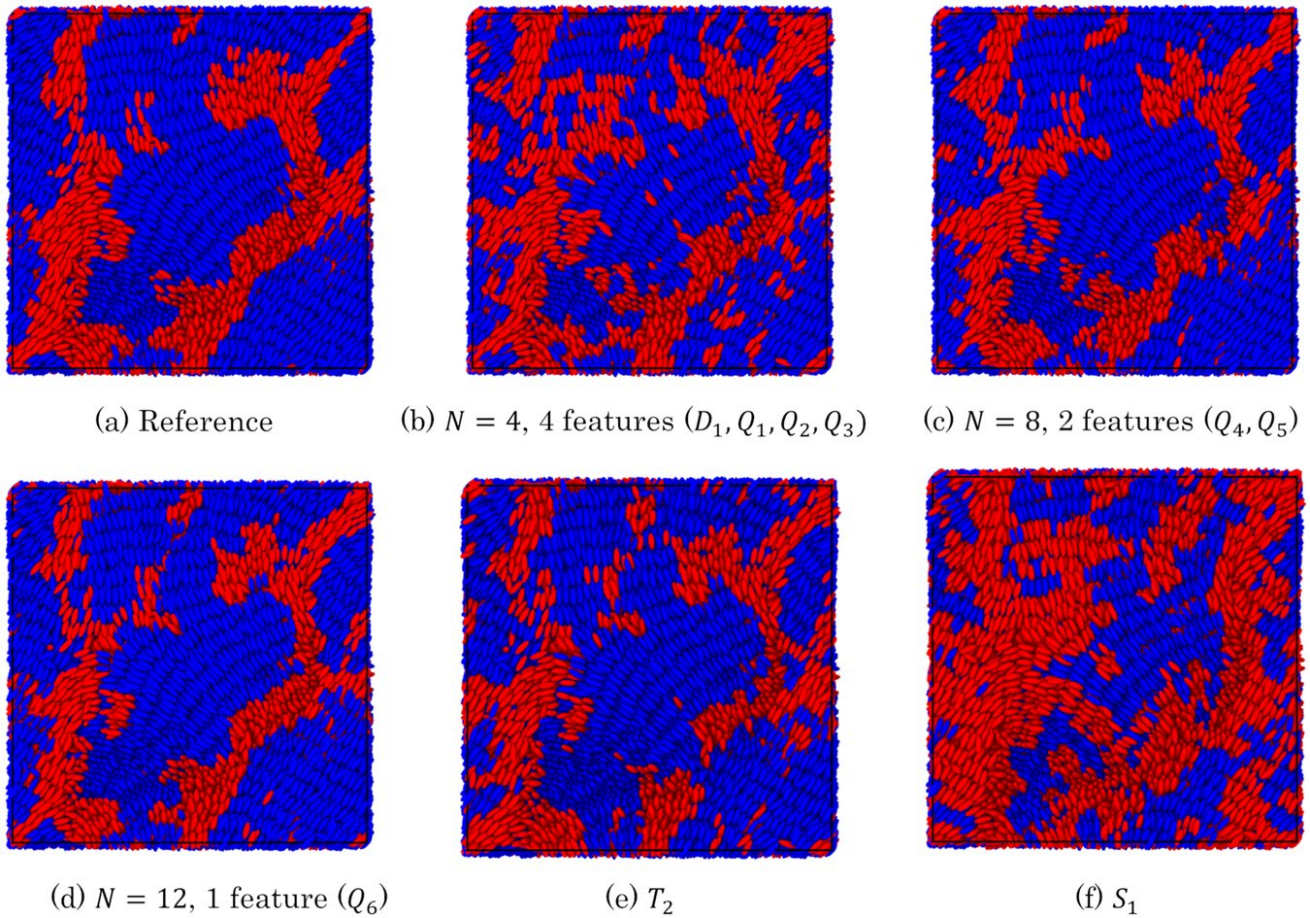
Results of local structure analysis using ML-LSA and conventional order parameter with (**a**) *N* = 27 (the reference), (**b**) *N* = 4, (**c**) *N* = 8, (**d**) *N* = 12, (**e**) local McMillan order parameter *T* with the parameter *d* = 3.0 and (**f**) local Onsager order parameter *S*. For *N* = 27, 17120 of the 62204 mesogens are in nematic-like structures. In contrast to Fig. 8, the classification of local structures was significantly improved. The clear alignments of smectic-like layers of blue-colored particles were visually acquired, while loose parallel alignments of nematic-like layers of red-colored particles were also seen. For *N* = 4, the four-feature ML model was used based on the results for monodomain systems. The visualized structure contains many small domains that do not correspond to the reference condition. For *N* = 8, the two-feature ML model was used. The small domains observed with *N* = 4 are less visible. For *N* = 12, the one-feature ML model was used. The domain shapes are quite similar to those of the reference structure. The local McMillan order parameter *T* achieved the adequacy similar to the two feature model with *N* = 8. The local Onsager order parameter *S* could not distinguish local structures properly. Definitions of the above order parameters are shown in the Supporting Information.

Instead of conventional methods, the ML model constructed using the monodomain LCP systems was applied to the local structure analysis of complex LCP systems. For monodomain systems, nematic- or smectic-like trajectories themselves can be used as the supervisor. However, this is difficult in complex systems including nematic- and smectic-like mesogenic structures. Thus, the ML model determined from monodomain systems were applied as a reliable function to distinguish local structures. The classification accuracy was evaluated from the “consistency” of the classification results for each local structure. First, the results of the most “accurate” analysis were obtained from using all the ML models determined under the largest condition of *N* = 27. This was then used as a reference for evaluating the “consistency” of the results under the computationally less costly conditions attained by the reduction of *N* and number of features. Next, the analyses were performed for small values of *N* using small number of features. The correct answer rate with respect to the reference was calculated as the index of consistency. Namely, the index of consistency *X*_c_ was defined as follows,

4 $${X}_{{\rm{c}}}=\frac{{Z}_{c,\mathrm{correct}}}{{Z}_{c,\mathrm{total}}},$$

where the *Z*_c,correct_ is the number of correct answers with respect to the reference, and *Z*_c,total_ is the number of total answers. Figure 9 visualizes the results of local structure analyses for *N* = 27 (the reference), *N* = 4, *N* = 8, and *N* = 12. For *N* = 27, 17120 of the 62204 mesogens were found to be in nematic-like structures. In comparison with the results of four conventional methods in Fig. 8, the classification of local structures was significantly improved. More specifically, the clear alignments of smectic-like layers of blue-colored particles were visually acquired, while loose parallel alignments of nematic-like layers of red-colored particles were also seen. For *N* = 4, the four-feature ML model was used based on the results for monodomain systems. The visualized structure contained numerous small domains that did not correspond to the reference condition. The *X*_c_ was 0.82, indicating poor consistency. Generally, structure classification requires a correct answer rate of at least 0.9. For *N* = 8, the two-feature ML model was used. The small domains observed with *N* = 4 were less visible. The *X*_c_ was 0.89, indicating adequate consistency. For *N* = 12, the one-feature ML model was used. The domain shapes were quite similar to those in the reference structure. The *X*_c_ was 0.92, indicating good consistency. Considering the good performance of McMillan order parameter to distinguish monodomain nematic- and smectic-like structures shown in Fig. 2, one big question still remains: can the local McMillan order parameter *T* classify the complex local structures of LCPs with high accuracy? The results of *T* was similar to that of two-feature ML model with *N* = 8. The *X*_c_ was 0.88, indicating adequate consistency. Therefore *Q*_6_ was better than *T*. Overall, *Q*_6_ was found to be the best order parameter for classifying the local structures of complex LCP systems. The values of index of consistency for all cases are presented in Table 2.

**Table 2:** Correct answer rate of feature-combined models with respect to the reference condition.

Number of neighborhood particles	Number of features	Consistency
*N* = 4	4 (*D*_1_, *Q*_1_, *Q*_2_, *Q*_3_)	0.82
*N* = 5	4 (*D*_2_, *Q*_7_, *Q*_8_, *T*_1_)	0.85
*N* = 6	4 (*D*_3_, *D*_4_, *Q*_9_, *Q*_10_)	0.87
*N* = 8	2 (*Q*_4_, *Q*_5_)	0.89
*N* = 12	1 (*Q*_6_)	0.92
*N* = 12	1 (*T*_2_)	0.88
*N* = 12	1 (*S*_1_)	0.63

The definitions of the order parameters *D*_1_–*D*_4_, *Q*_1_–*Q*_10_, *S*_1_, *T*_1_ and *T*_2_ are shown in the Supporting Information.

Note that the above analyses were performed for three different trajectories quenched from three different initial coordinates, and the results were almost the same in all cases.

## Conclusion

We have developed a Machine Learning-aided Local Structure Analyzer (ML-LSA) to classify the structures of LCPs containing rigid ellipsoidal particles. The ML model for local structure analyses was determined from small, and thus computationally efficient, monodomain LCP trajectories. The ML model was found to distinguish between nematic- and smectic-like monodomain structures with high accuracy. Furthermore, using feature selection, a single or set of order parameter(s) with good classification performance was revealed. For monodomain LCP trajectories, ML-LSA can distinguish between two structures using only the coordinates of the 1–4 nearest particles, which contain very little information about local structures.

ML-LSA was applied to the local structure analyses of large and complex quenched LCP trajectories. The quenched trajectories exhibited complex local structures containing nematic- and smectic-like mesogenic structures. As shown in Fig. 8, conventional particle-based local structure analyzing methods could not distinguish between these structures. However, we successfully classified these complex local structures using ML-LSA, regardless of the user-dependent parameter, i.e., the aspect ratio of mesogenic ellipsoids. Furthermore, the results of ML-LSA suggest that the best order parameter is *Q*_6_ for distinguishing nematic- from smectic-like mesogenic structures. For complex quenched LCP trajectories, ML-LSA can distinguish the two structures using the coordinates of the 1–12 nearest particles.

The above analyses were performed automatically, systematically, and independent of previous knowledge using ML. This means that ML-LSA can overcome the difficulty of manually identifying the specific order parameter for the classification of complex structures. ML-LSA has strong potential for classifying the structures of other complex chemical and biological molecular systems. In future studies, the capabilities of ML-LSA will be intensively evaluated.

## Supplementary information

Supplementary information
