# Supplementary material for: Machine learning-aided analysis for complex local structure of liquid crystal polymers
Source: Sci Rep. 2019 Nov 8;9:16370. doi: 10.1038/s41598-019-51238-1 (PMC6841663; doi:10.1038/s41598-019-51238-1)
Supplement: Supplementary file 1 — Supplementary information [file 41598_2019_51238_MOESM1_ESM.pdf]

Supporting Information for "Machine Learning-Aided Analysis for Complex Local Structure of Liquid Crystal Polymers"

Hideo Doi, Kazuaki Z. Takahashi, and Takeshi Aoyagi

Research Center for Computational Design of Advanced Functional Materials, National Institute of Advanced Industrial Science and Technology (AIST), Central 2, 1-1-1 Umezono, Tsukuba-shi, Ibaraki 305-8568, Japan

Kenji Tagashira

Research Association of High-Throughput Design and Development for Advanced Functional Materials, Central 2, 1-1-1 Umezono, Tsukuba-shi, Ibaraki 305-8568, Japan

Jun-ichi Fukuda

Department of Physics, Faculty of Science, Kyushu University, 744 Motooka, Nishi-ku, Fukuoka-shi, Fukuoka 819-0395, Japan

A. Basic Variables for Order Parameters of Rigid Ellipsoidal Particles

To ensure that the order parameters are useful for rigid ellipsoidal particles, the main problem is determining expressions for their coordinates and orientation. We defined the coordinate $\boldsymbol{r}_i^{(o_i)}$ of ellipsoidal particle i as

$$\boldsymbol{r}_i^{(o_i)} = \boldsymbol{r}_i + o_i \boldsymbol{u}_i \tag{1}$$

where $\boldsymbol{r}_i$ is the center position of particle i, $\boldsymbol{u}_i$ is the unit direction vector of particle i, and o_i is the factor expressing the center position or each end of the direction vector of particle i. We also defined an array of o_i, $\boldsymbol{o}_i$. If the center position and both ends of the direction vector for particle i can be expressed using $o_i = 0$, 1, or -1, respectively, $\boldsymbol{o}_i$ becomes $(0, 1, -1)$. The term $\boldsymbol{r}_{ij}^{(o_i,o_j)}$ is the position vector from $\boldsymbol{r}_i^{(o_i)}$ to $\boldsymbol{r}_j^{(o_j)}$.

For local structure analyses, a basic definition expressing local information around particle i is required. We defined an array of identification numbers for each particle around i, $\boldsymbol{N}_\mathrm{b}(i)$, containing pure local information. Inside $\boldsymbol{N}_\mathrm{b}(i)$, the identification numbers were stored in ascending order of distance from i. n_b is the number of nearest neighbors of i, and is equal to the total count of the number of identification numbers stored in $\boldsymbol{N}_\mathrm{b}(i)$. We also defined $\tilde{\boldsymbol{N}}_\mathrm{b}(i)$, which is $\boldsymbol{N}_\mathrm{b}(i)$ plus the identification number of i. Then, $\tilde{n}_\mathrm{b}$ is equal to $n_\mathrm{b} + 1$.

Local information between particles i and j was also required for a certain kind of order parameter. We defined an array of identification numbers of each particle around i and j, $\boldsymbol{N}_\mathrm{b}(i, j)$. Candidates that should be stored in $\boldsymbol{N}_\mathrm{b}(i, j)$ were selected from the particles stored in $\boldsymbol{N}_\mathrm{b}(i)$ and $\boldsymbol{N}_\mathrm{b}(j)$. For each candidate, a degree of importance was determined as local information from the length perpendicular from the coordinates of the candidate particle to an axis on the vector $\boldsymbol{r}_{ij}^{(o_i,o_j)}$. Inside $\boldsymbol{N}_\mathrm{b}(i, j)$, identification numbers were stored in ascending order of the length of this perpendicular. m_b is the number of nearest neighbors between i and j, and is equal to the total count of the number of identification numbers stored in $\boldsymbol{N}_\mathrm{b}(i, j)$.

B. Local Onsager Order Parameter

We modified Onsager's original order parameter[1] for local structure analyses. The local Onsager order parameter $S_i^{(N,a)}$ is defined as follows:

$$S_i^{(N,a)} = \frac{1}{\tilde{n}_\mathrm{b}} \sum_{j \in \tilde{\boldsymbol{N}}_\mathrm{b}(i)} S_j^{(N,a-1)}, \tag{2}$$

$$S_i^{(N,1)} = \frac{\sum_{j \in \boldsymbol{N}_\mathrm{b}(i)} \{3(\boldsymbol{u}_i \cdot \boldsymbol{u}_j)^2 - 1\}}{2n_\mathrm{b}}. \tag{3}$$

Note that Eq. (2) expresses an averaging operation of the values of local order parameter that particles neighboring the i-th particle have. The a is the number of times of the averaging, and thus the Eq. (2) is a recurrence formula of a.

C. Local McMillan Order Parameter

We modified the order parameter suggested by McMillan[2]. The local McMillan order parameter $T_i^{(N,a,o_i,o_j)}$ is defined as follows:

$$T_i^{(N,a,o_i,o_j)} = \frac{1}{\tilde{n}_\mathrm{b}} \sum_{j \in \tilde{\boldsymbol{N}}_\mathrm{b}(i)} T_j^{(N,a-1,o_i,o_j)}, \tag{4}$$

$$T_i^{(N,1,o_i,o_j)} = \frac{\sum_{j \in \boldsymbol{N}_\mathrm{b}(i)} \cos(2\pi z/d)\{3(\boldsymbol{u}_i \cdot \boldsymbol{u}_j)^2 - 1\}}{2n_\mathrm{b}}, \tag{5}$$

where z is the distance from the coordinates of j to the plane passing through the coordinates of i and is normal to the orientation vector of i, and d is represents the distance between two layers of a smectic-A structure.

D. Bond Order Parameter

We modified the order parameter suggested by Steinhardt *et al.*[3]. Note that Lechner *et al.* proposed a method that improves the performance of the original order parameter by averaging the spherical harmonic functions of neighborhood particles[4]. The bond order

parameter $Q_i^{(N,l,a,b,o_i,o_j,p)}$ is defined as follows:

$$Q_i^{(N,l,a,b,o_i,o_j,p)} = \frac{1}{\tilde{n}_\mathrm{b}} \sum_{j \in \tilde{\boldsymbol{N}}_\mathrm{b}(i)} Q_j^{(N,l,a-1,b,o_i,o_j,p)} \tag{6}$$

$$Q_i^{(N,l,1,b,o_i,o_j,p)} = \sqrt{\frac{4\pi}{2l+1} \sum_{m=-l}^{l} |q_{lm}^{(N,l,1,b,o_i,o_j,p)}(i)|^2} \tag{7}$$

$$q_{lm}^{(N,l,1,b,o_i,o_j,p)}(i) = \frac{1}{\tilde{n}_\mathrm{b}} \sum_{j \in \tilde{\boldsymbol{N}}_\mathrm{b}(i)} q_{lm}^{(N,l,1,b-1,o_i,o_j,p)}(j) \tag{8}$$

$$q_{lm}^{(N,l,1,1,o_i,o_j,p)}(i) = \frac{1}{n_\mathrm{b}} \sum_{j \in \boldsymbol{N}_\mathrm{b}(i)} Y_{lm}(\theta_{ij}^{(o_i,o_j)}, \phi_{ij}^{(o_i,o_j)}) + p \sum_{o^* \neq o_i} Y_{lm}(\theta_{ii}^{(o_i,o^*)}, \phi_{ii}^{(o_i,o^*)}) \tag{9}$$

where l is a spherical harmonic parameter, b is the number of times averaging is applied to the spherical harmonic functions, p is a parameter for weighting the orientation vector of i, $Y_{lm}(\theta, \phi)$ is the spherical harmonic function, and o^* is an element of $\boldsymbol{o}_i$.

Note that $Q_i^{(N,l,a=1,b=1,o_i=0,o_j=0,o_k=0,p=0)}$ is equal to Steinhardt's order parameter[3] and $Q_i^{(N,l,a=2,b=2,o_i=0,o_j=0,o_k=0,p=0)}$ is equal to Lechner's order parameter[4].

E. Common Neighborhood Parameter

We modified the order parameter suggested by Radhi *et al.*[5]. Note that this type of order parameter was originally developed by Honeycutt *et al.*[6], but was not quantified. The quantified version was first developed by Tsuzuki *et al.*[7], and then extended by Radhi *et al.*[5]. The common neighborhood parameter $A_i^{(N,a,m_\mathrm{b},o_i,o_j,o_k)}$ is defined as follows:

$$A_i^{(N,a,m_\mathrm{b},o_i,o_j,o_k)} = \frac{1}{\tilde{n}_\mathrm{b}} \sum_{j \in \tilde{\boldsymbol{N}}_\mathrm{b}(i)} A_j^{(N,a-1,m_\mathrm{b},o_i,o_j,o_k)}, \tag{10}$$

$$A_i^{(N,1,m_\mathrm{b},o_i,o_j,o_k)} = \frac{1}{n_\mathrm{b}} \sum_{j \in \boldsymbol{N}_\mathrm{b}(i)} | \sum_{k \in \boldsymbol{N}_\mathrm{b}(i,j)} (\boldsymbol{r}_{ik}^{(o_i,o_k)} + \boldsymbol{r}_{jk}^{(o_j,o_k)})|^2. \tag{11}$$

F. Predominant Common Neighborhood Parameter

We modified the order parameter suggested by Radhi *et al.*[5]. The predominant common neighborhood parameter $P_i^{(N,a,m_\mathrm{b},o_i,o_j,o_k)}$ is defined as follows:

$$P_i^{(N,a,m_\mathrm{b},o_i,o_j,o_k)} = \frac{1}{\tilde{n}_\mathrm{b}} \sum_{j \in \tilde{\boldsymbol{N}}_\mathrm{b}(i)} P_j^{(N,a-1,m_\mathrm{b},o_i,o_j,o_k)}, \tag{12}$$

$$P_i^{(N,1,m_\mathrm{b},o_i,o_j,o_k)} = \frac{1}{n_\mathrm{b}} \sum_{j \in \boldsymbol{N}_\mathrm{b}(i)} | \sum_{k \in \boldsymbol{N}_\mathrm{b}(i,j)} (\boldsymbol{r}_{ik}^{(o_i,o_k)} + \boldsymbol{r}_{kj}^{(o_j,o_k)})|^2. \tag{13}$$

G. Another Predominant Common Neighborhood Parameter

We modified the order parameter suggested by Radhi *et al.*[5]. The second predominant common neighborhood parameter $M_i^{(N,a,m_\mathrm{b},o_i,o_j,o_k)}$ is defined as follows:

$$M_i^{(N,a,m_\mathrm{b},o_i,o_j,o_k)} = \frac{1}{\tilde{n}_\mathrm{b}} \sum_{j \in \tilde{\boldsymbol{N}}_\mathrm{b}(i)} M_j^{(N,a-1,m_\mathrm{b},o_i,o_j,o_k)}, \tag{14}$$

$$M_i^{(N,1,m_\mathrm{b},o_i,o_j,o_k)} = \frac{1}{n_\mathrm{b}} | \sum_{j \in \boldsymbol{N}_\mathrm{b}(i)} \sum_{k \in \boldsymbol{N}_\mathrm{b}(i,j)} (\boldsymbol{r}_{ij}^{(o_i,o_k)} + \boldsymbol{r}_{kj}^{(o_j,o_k)})|^2. \tag{15}$$

H. Bond Angle Analysis

Bond angle analysis was originally suggested by Ackland *et al.*[8], but was not quantified. We quantified and modified this analysis to give the following order parameter:

$$B_i^{(N,a,\alpha,\kappa,\phi,o_i,o_j,o_k)} = \frac{1}{\tilde{n}_\mathrm{b}} \sum_{j \in \tilde{\boldsymbol{N}}_\mathrm{b}(i)} B_j^{(N,a-1,\alpha,\kappa,\phi,o_i,o_j,o_k)}, \tag{16}$$

$$B_i^{(N,1,\alpha,\kappa,\phi,o_i,o_j,o_k)} = \frac{1}{n_\mathrm{b}(n_\mathrm{b}-1)} \sum_{j,k \in \boldsymbol{N}_\mathrm{b}(i),\, j \neq k} f^{(\alpha,\kappa,\phi,o_i,o_j,o_k)}(\theta_{jik}^{(o_i,o_j,o_k)}), \tag{17}$$

$$f^{(\alpha,\kappa,\phi,o_i,o_j,o_k)}(\theta_{jik}) = \cos^\kappa(\alpha\theta_{jik}^{(o_i,o_j,o_k)} + \varphi), \tag{18}$$

where α is the angle factor, κ is the number of exponents for the cosine function, φ is the angle offset, and $\theta_{ijk}^{(o_i,o_j,o_k)}$ is the angle between vectors $\boldsymbol{r}_{ij}^{(o_i,o_j)}$ and $\boldsymbol{r}_{ik}^{(o_i,o_k)}$.

I. Centrosymmetry Parameter Analysis

We modified the order parameter suggested by Kelchner *et al.*[9]. The centrosymmetry parameter analysis $C_i^{(N,a,o_i,o_j,o_k)}$ is defined as follows:

$$C_i^{(N,a,o_i,o_j,o_k)} = \frac{1}{\tilde{n}_\mathrm{b}} \sum_{j\in\tilde{\boldsymbol{N}}_\mathrm{b}(i)} C_j^{(N,a-1,o_i,o_j,o_k)} \tag{19}$$

$$C_i^{(N,1,o_i,o_j,o_k)} = \sum_{j=1}^{n_\mathrm{b}/2} |\boldsymbol{r}_{ij}^{(o_i,o_j)} + \boldsymbol{r}_{ik}^{(o_i,o_k)}|^2 \qquad (k = j + n_\mathrm{b}/2). \tag{20}$$

J. Neighbor Distance Analysis

Neighbor distance analysis was originally suggested by Stukowski[10], but was not quantified. We quantified and modified this analysis to give the following order parameter:

$$D_i^{(N,a,o_i,o_j,o_k,f_1,f_2,f_3)} = \frac{1}{\tilde{n}_\mathrm{b}} \sum_{j\in\tilde{\boldsymbol{N}}_\mathrm{b}(i)} D_j^{(N,a-1,o_i,o_j,o_k,f_1,f_2,f_3)}, \tag{21}$$

$$D_i^{(N,1,o_i,o_j,o_k,f_1,f_2,f_3)} = \frac{1}{n_b(n_\mathrm{b}-1)} \sum_{j,k\in\boldsymbol{N}_\mathrm{b}(i),\, j\neq k} f_1(\boldsymbol{r}_{ij}^{(o_i,o_j)}) f_2(\boldsymbol{r}_{ik}^{(o_i,o_k)}) f_3(\boldsymbol{r}_{jk}^{(o_j,o_k)}). \tag{22}$$

where f_1, f_2, and f_3 are functions that depend on the inter-particle distance. The possible functions for f_1, f_2, and f_3 are listed in Table S1.

K. Angular Fourier-Series-like Parameter

We modified the order parameter suggested by Bartk *et al.* as the descriptor of the chemical environment[11, 12]. The angular Fourier-series-like parameter $F_i^{(N,a,o_i,o_j,o_k,f_1,f_2,\beta)}$ is defined as follows:

$$F_i^{(N,a,o_i,o_j,o_k,f_1,f_2,\beta)} = \frac{1}{\tilde{n}_\mathrm{b}} \sum_{j\in\tilde{\boldsymbol{N}}_\mathrm{b}(i)} F_j^{(N,a-1,o_i,o_j,o_k,f_1,f_2,\beta)}, \tag{23}$$

$$F_i^{(N,1,o_i,o_j,o_k,f_1,f_2,\beta)} = \frac{1}{n_\mathrm{b}(n_\mathrm{b}-1)} \sum_{j,k\in\boldsymbol{N}_\mathrm{b}(i),\, j\neq k} \tag{24}$$

$$f_1(\boldsymbol{r}_{ij}^{(o_i,o_j)}) f_2(\boldsymbol{r}_{ik}^{(o_i,o_k)}) \cos(\beta\theta_{jik}^{(o_j,o_i,o_k)}), \tag{25}$$

where β is the angle factor.

L. Angle Histogram Analysis Parameter

We developed the angle histogram analysis parameter. When considering combinations of triangles for particles i, j, and k in a system, $\theta_{ijk}^{(o_i,o_j,o_k)}$ should have a histogram $h_i^{(N,1,\lambda,o_i,o_j,o_k)}$ at $0 \leq \theta_{ijk}^{(o_i,o_j,o_k)} \leq \pi$, where λ is the number of segments of the histogram. For local structure analysis, $h_i^{(N,1,\lambda,o_i,o_j,o_k)}$ was averaged over the neighbor particles as follows:

$$h_i^{(N,a,\lambda,o_i,o_j,o_k)} = \frac{1}{\tilde{n}_{\rm b}(i)} \sum_{j \in \tilde{N}_b(i)} h_j^{(N,a-1,\lambda,o_i,o_j,o_k)}. \tag{26}$$

The angle histogram analysis parameter $H_i^{(N,a,\lambda,o_i,o_j,o_k)}$ was then defined as follows:

$$H_i^{(N,a,\lambda,o_i,o_j,o_k,\nu)} = FT_{\rm ampl.}(h_i^{(N,a,\lambda,o_i,o_j,o_k)})\delta(\tau - \nu), \tag{27}$$

where ν is the frequency offset of Dirac's delta function, $FT_{\rm ampl.}$ is a function expressing the amplitude computed from Fourier analysis, δ is Dirac's delta function, and τ is the frequency of Dirac's delta function.

M. Variables for Order Parameters

For convenience, details of the variables used in the eleven order parameter equations described above are presented in Table S1. As stated in Section 2.1 of the article, the total number of order parameters, i.e., the number of the combinations of all possible values in the last column of Table S1, is approximately 1,600,000. Some of these variables are continuous, so that the number of possible conditions can be much larger than 1,600,000 when a finer choice of such continuous variables is made. However, almost all of such attempts could not increase the correct answer rate for classification of liquid crystal polymer systems in this work. Note that we initially screened such continuous variables to determine the choices show in Table S1.

TABLE S1. Variables used in the order parameter equations

Variables	Description	Order param.	Values or functions
N	number of neighborhood particles	all	4, 6, 8, 10, 12, 16, 18, 24, 27
R	radii of neighborhood particles	all	1.75, 2.00, 2.25, 2.50, 2.75, 3.00
a	number of times averaging applied	all	1, 2
$\boldsymbol{o}_i$	[a]	all	(0, 0.5, -0.5), (0, 1.0, -1.0), (0, 1.5, -1.5)
d	distance between two smectic layers	T	0.75, 1.0, 1.5, 2.0, 2.5, 3.0, 3.5
b	[b]	Q	1,2
l	parameter for spherical harmonics	Q	2, 3, 4, 6, 8
p	weighting factor for direction vector	Q	0, 0.5, 1, $N/2$, N
m_{b}	[c]	A, P, M	2, 3, 4, 5
α	angle factor	B	1, 2, 3, 4, 6
φ	angle offset	B	$0, \frac{2\pi}{3}, \frac{\pi}{2}, \frac{\pi}{3}, \frac{\pi}{4}, \frac{\pi}{5}, \frac{\pi}{6}, \frac{\pi}{8}, \frac{\pi}{9}, \frac{\pi}{12}$
κ	number of exponents for cosine func.	B	1, 2, 3, 4
f_1,f_2,f_3	distance-dependent functions	D, F	$r, r^2, \frac{1}{r}, \frac{1}{r^2}, 1-\exp\{-\frac{(r-1.75)^2}{2\times0.15^2}\}$, $[1+\exp\{-\frac{(r-1.75)^2}{2\times0.15^2}\}]/2$
β	angle factor	F	1, 2, 3, 4, 5, 6
λ	number of segments in histogram	H	12,24,36
ν	frequency offset	H	3, 4, 5, 6, 8

[a] Factor expressing the center position or each end of direction vector of the particle i

[b] Number of times averaging applied to spherical harmonic functions

[c] Number of nearest neighbors between i and j, equal to the total count of the number of identification numbers stored in $\boldsymbol{N}_{\mathrm{b}}(i,j)$

N. Order parameters in Figure 6 and Table 2

$$D_1 = D^{N=4,a=2,o_i=0,o_j=1,o_k=0,f_{ij}=1/r,f_{ik}=r^2,f_{jk}=r} \tag{28}$$

$$D_2 = D^{N=5,a=2,o_i=0.5,o_j=-0.5,o_k=0,f_{ij}=\frac{1}{r^2},f_{jk}=r,f_{ik}=r^2} \tag{29}$$

$$D_3 = D^{N=6,a=2,o_i=0,o_j=-0.5,o_k=0,f_{ij}=[1+\exp\{-\frac{(r-1.75)^2}{2\times0.15^2}\}]/2,f_{jk}=r,f_{ik}=1-\exp\{-\frac{(r-1.75)^2}{2\times0.15^2}\}} \tag{30}$$

$$D_4 = D^{N=6,a=2,o_i=0,o_j=0,o_k=0.5,f_{ij}=[1+\exp\{-\frac{(r-1.75)^2}{2\times0.15^2}\}]/2,f_{jk}=\frac{1}{4},f_{ik}=\frac{1}{r^2}} \tag{31}$$

$$Q_1 = Q^{N=4,l=4,a=2,b=2,o_i=1,o_j=0,p=0} \tag{32}$$

$$Q_2 = Q^{N=4,l=4,a=2,b=2,o_i=0,o_j=0,p=1} \tag{33}$$

$$Q_3 = Q^{N=4,l=6,a=1,b=2,o_i=0.5,o_j=1,p=0.5} \tag{34}$$

$$Q_4 = Q^{N=8,l=4,a=2,b=2,o_i=1,o_j=0,p=0} \tag{35}$$

$$Q_5 = Q^{N=8,l=6,a=2,b=2,o_i=0,o_j=0,p=1} \tag{36}$$

$$Q_6 = Q^{N=12,l=2,a=2,b=2,o_i=0,o_j=0,p=0} \tag{37}$$

$$Q_7 = Q^{N=5,l=2,a=2,b=2,o_i=0,o_j=0,p=0} \tag{38}$$

$$Q_8 = Q^{N=5,l=6,a=2,b=2,o_i=0,o_j=-0.5,p=0.5} \tag{39}$$

$$Q_9 = Q^{N=6,l=2,a=2,b=2,o_i=0,o_j=0,p=0} \tag{40}$$

$$Q_{10} = Q^{N=6,l=6,a=2,b=2,o_i=0,o_j=-0.5,p=0} \tag{41}$$

$$S_1 = S^{N=12,a=2} \tag{42}$$

$$T_1 = T^{N=5,a=2,z=2.0} \tag{43}$$

$$T_2 = T^{N=12,a=2,z=3.0} \tag{44}$$

[1] L. Onsager, Annals of the New York Academy of Sciences **51**, 627 (1949).

[2] W. McMillan, Physical Review A **4**, 1238 (1971).

[3] P. J. Steinhardt, D. R. Nelson, and M. Ronchetti, Physical Review B **28**, 784 (1983).

[4] W. Lechner and C. Dellago, Journal of Chemical Physics **129**, 114707 (2008), arXiv:arXiv:0806.3345v1.

[5] A. Radhi and K. Behdinan, Computational Materials Science **126**, 182 (2017).

[6] J. D. Honeycutt and H. C. Andersen, The Journal of Physical Chemistry **91**, 4950 (1987).

[7] H. Tsuzuki, P. S. Branicio, and J. P. Rino, Computer Physics Communications **177**, 518 (2007).

[8] G. J. Ackland and A. P. Jones, Physical Review B - Condensed Matter and Materials Physics **73**, 1 (2006).

[9] C. L. Kelchner, S. J. Plimpton, and J. C. Hamilton, Physical Review B **58**, 11085 (1998).

[10] A. Stukowski, Modelling and Simulation in Materials Science and Engineering **20**, 045021 (2012), arXiv:1202.5005.

[11] A. P. Bartók, R. Kondor, and G. Csányi, Physical Review B - Condensed Matter and Materials Physics **87**, 1 (2013), arXiv:1209.3140.

[12] A. Seko, A. Togo, and I. Tanaka, in *Nanoinformatics* (Springer Singapore, Singapore, 2018) pp. 3–23, arXiv:1709.01666.
